# Linking COVID-19 and Cardiovascular Risk: The Diagnostic Potential of Asymmetric Dimethylarginine, Neopterin, and Vitamin D in Coronary Artery Disease

**DOI:** 10.7759/cureus.91814

**Published:** 2025-09-08

**Authors:** Gizem Uncu, Gülçin Alp Avci, Mustafa Eren, Emre Avci

**Affiliations:** 1 Molecular Biology and Genetics, Hitit University Institute of Graduate Education, Corum, TUR; 2 Basic Medical Sciences, Medical Microbiology, University of Health Sciences (Gulhane Campus), Ankara, TUR; 3 Pulmonology, Corum Chest Diseases Hospital, Corum, TUR; 4 Biochemistry, University of Health Sciences (Gulhane Campus), Ankara, TUR

**Keywords:** adma, biomarkers, coronary artery disease, covid-19, neopterin, vaccination, vitamin d

## Abstract

Objective: Coronavirus disease 2019 (COVID-19) is associated with more severe clinical outcomes and increased mortality, particularly in individuals with comorbidities, such as hypertension and diabetes. This study aimed to evaluate the risk of developing coronary artery disease (CAD) about COVID-19 infection and vaccination status by measuring the serum levels of asymmetric dimethylarginine (ADMA), neopterin (NP), and vitamin D.

Materials and methods: A total of 120 volunteers aged 30-65 years, presenting to a Chest Diseases Outpatient Clinic, were enrolled and categorized into four groups: COVID-19(+) vaccinated; COVID-19(+) unvaccinated; COVID-19(-) vaccinated; and COVID-19(-) unvaccinated. Routine biochemical parameters were assessed using an autoanalyzer. Serum ADMA and NP levels were quantified via enzyme-linked immunosorbent assay (ELISA), while vitamin D levels were obtained using a chromatography-based method.

Results: According to the findings of our study, the serum levels of both ADMA and NP were elevated in individuals with COVID-19. While the increase in ADMA was statistically significant (p<0.05), the increase in NP was not (p>0.05). Furthermore, the lower, though not significant, vitamin D observed in COVID-19-positive participants is considered a significant finding. However, higher vitamin D was observed in vaccinated individuals compared to unvaccinated individuals, which was not statistically significant (p>0.05).

Discussion and conclusion: The side effects of COVID-19 infection and vaccination continue to be a significant concern for all societies. Our study examined the association with the increased risk of CAD associated with this process. Our findings suggest that COVID-19 infection potentially influences CAD risk by affecting inflammatory and endothelial biomarkers, such as ADMA and NP. Simultaneous assessment of these markers can provide valuable information for cardiovascular risk profiling. Vaccination appears protective; it helps maintain higher vitamin D levels and is associated with a more controlled immune response, rather than increased risk. Including vitamin D status in this assessment may further clarify the interaction between immune function and vascular health.

## Introduction

Coronavirus disease 2019 (COVID-19) has become a major public health concern as a rapidly spreading infectious disease with high morbidity and mortality worldwide [1]. Particularly in individuals with chronic conditions, such as cardiovascular diseases, the more severe course of COVID-19 and the increased risk of complications pose significant challenges in clinical management [2]. During the COVID-19 pandemic, over 45 million cases and approximately 1.2 million deaths have been reported globally. Although the transmission rate between individuals is higher than that of SARS-CoV, the mortality rate is relatively lower. The disease can cause multisystemic failure by affecting multiple organs, including primarily the respiratory system, as well as the liver, kidneys, gastrointestinal system, heart, and central nervous system [3]. It has been defined as a complex disease affecting not only the respiratory system but also multiple organ systems, particularly the cardiovascular system. In patients with comorbidities, such as diabetes, hypertension, obesity, advanced age, and coronary artery disease (CAD), the disease tends to progress more severely and is associated with an increased risk of mortality [4,5]. Individuals with a prior history of CAD, cerebrovascular disease, or stroke are at a higher risk for severe disease and death due to COVID-19 [6].

The endothelium plays a central role in maintaining vascular functions, with nitric oxide (NO) being the primary mediator of this regulation [7]. Asymmetric dimethylarginine (ADMA), an endogenous inhibitor of NO synthase (NOS), reduces NO production, leading to endothelial dysfunction, which plays a significant role in the development of atherosclerotic cardiovascular diseases [8-10]. Elevated ADMA levels increase cardiovascular risk and mortality, and this effect is particularly exacerbated by smoking [11]. ADMA elevation is considered a critical biomarker in the pathophysiology of diseases such as hypertension and atherosclerosis [12,13].

Neopterin (NP) is a pteridine derivative secreted by monocytes-macrophages and activated dendritic cells and is considered a reliable marker of cellular immune activation [14,15]. NP accumulation induced by interferon-gamma (IFN-γ) affects tryptophan metabolism and reflects the systemic immune response, involving the enzyme indoleamine 2,3-dioxygenase (IDO) in the process [16]. NP also serves as a marker of oxidative stress, capable of increasing iNOS expression and NO production [17,18]. NP levels are significantly elevated in viral infections, autoimmune diseases, transplant rejection, and certain cancers [15,19]. Recent studies suggest that NP and its derivatives may play active roles in redox-sensitive metabolic pathways [20].

Vitamin D is a hormone with immunomodulatory effects on the immune system, capable of suppressing inflammation and reducing viral replication. It is synthesized in the skin via ultraviolet B radiation and plays a critical role in bone, immune, and cardiovascular health [21]. In Turkey, vitamin D deficiency is highly prevalent; levels below 20 ng/mL are considered deficient and may negatively impact cardiovascular health by disrupting parathyroid hormone (PTH) balance; vitamin supplementation has been shown to mitigate these effects. In the context of COVID-19, several studies have demonstrated the antiviral and anti-inflammatory roles of vitamin D. Meta-analyses have shown that severe deficiency increases the risk of mortality by approximately 1.9 times [22,23].

The primary objective of this study is to compare ADMA, NP, and vitamin D levels in individuals based on their history of COVID-19 infection and vaccination status and to evaluate the prognostic potential of these biomarkers in relation to CAD. Specifically, the study aims to determine whether COVID-19-related increases in ADMA and NP levels, when considered alongside vitamin D levels, may serve as predictive indicators of CAD risk, and whether this biomarker combination can provide clinical utility in risk assessment and patient management.

## Materials and methods

This study was planned at the Chest Diseases Hospital in Corum, Turkey. The primary aim of the study was to compare serum levels of ADMA, NP, and vitamin D across four subgroups defined by COVID-19 infection status and vaccination status and to evaluate the prognostic predictive value of these parameters in terms of CAD.

In our study, the number of participants in each group was determined as 30 according to the power analysis, and a hypothetical design based on the assumption of α = 0.05 significance level, 80% statistical power (1-β), and medium effect size (d ≈ 0.5) was preferred. This sample size was deemed appropriate and consistent with similar biomarker studies in the literature. Accordingly, the total sample size was set at 120 participants.

Ethical approval for the study was obtained from the Ethics Committee of Corum Chest Diseases Hospital (Decision No: 2023/157), and written informed consent was obtained from all participants.

Participant groups

The individuals included in the study were classified into the following four groups: Group I: COVID-19-positive and vaccinated (n = 30); Group II: COVID-19-positive and unvaccinated (n = 30); Group III: COVID-19-negative and vaccinated (n = 30); and Group IV: COVID-19-negative and unvaccinated (control group) (n = 30).

The COVID-19 infection status of participants was verified using medical records, PCR test results, and/or SARS-CoV-2 IgG antibody test results. Vaccination status was defined as having received at least one dose of a COVID-19 vaccine, with a minimum of 14 days having passed since administration

Participant selection criteria

Inclusion criteria: Voluntary male or female individuals aged between 30 and 65 years, with clearly defined COVID-19 infection and vaccination status, and no evidence of active systemic disease (e.g., malignancy, heart disease, diabetes, tuberculosis, etc.) based on clinical and laboratory findings.

Exclusion criteria: Individuals diagnosed with diabetes, cancer, or malignant hematologic disorders; those who were pregnant or breastfeeding; those with active infections (e.g., tuberculosis, typhoid, brucellosis, etc.); and those who did not sign the informed consent form.

Specific criteria for the control group: Group IV, composed of unvaccinated individuals with a negative COVID-19 IgG test result, included only those with no known history of any chronic disease.

Blood sample collection, processing, and biomarker analysis method

Venous blood samples were collected from participants after 12 hours of fasting. The samples were centrifuged at 4,000 g for 10 minutes. The obtained serum was collected under light-protected conditions and stored at -80 °C until the day of analysis [24]. Routine serum biochemistry tests were performed on the same day using an automated analyzer.

The primary objective of the study was to evaluate the prognostic significance of serum ADMA, NP, and vitamin D levels in individuals who had previously been infected with COVID-19, with particular focus on their potential as cardiovascular risk biomarkers following COVID-19 infection. Accordingly, serum levels of ADMA (as an indicator of endothelial dysfunction) and NP (as a marker of cellular immunity) were analyzed using the ELISA method, while vitamin D levels were measured using a chromatography-based method.

Statistical analysis

The study included four groups, each consisting of 30 participants, based on a priori power analysis. Continuous variables were expressed as mean ± standard deviation (SD). The normality of data distribution was assessed using the Kolmogorov-Smirnov test. For variables with a normal distribution, between-group comparisons were made using the Independent Samples t-test, and variance homogeneity was assessed using Levene’s test. Parametric comparisons of ADMA and NP levels were performed using the independent samples t-test. For vitamin D levels, which did not follow a normal distribution, the non-parametric Kruskal-Wallis test was applied. The significance level of p<0.05 was considered statistically significant. All analyses were conducted using Statistical Product and Service Solutions (SPSS, version 25.0; IBM SPSS Statistics for Windows, Armonk, NY).

## Results

In our study, markers of inflammation and oxidative stress were elevated in all COVID-19-positive groups. According to the Kolmogorov-Smirnov and Shapiro-Wilk normality tests, ADMA and NP levels were found to be normally distributed (p<0.05), while vitamin D levels were not. Based on these data, significance levels were examined using parametric and nonparametric tests.

ADMA and NP levels

According to the findings of our study, both ADMA and NP serum levels were found to be elevated in individuals with a history of COVID-19. The highest ADMA level was observed in the COVID+/vaccinated+ group (5.60 ± 1.77 µmol/L), while the lowest level was found in the COVID-/unvaccinated group (4.75 ± 0.97 µmol/L). The increase in ADMA levels was statistically significant (p=0.001; p<0.05). However, the increase in NP levels was not significant (p=0.847; p>0.05). This suggests that COVID-19 infection may increase ADMA levels and that vaccination does not alter this effect. The increase in ADMA levels observed in COVID-19 patients indicates an increased risk of CAD in these patients (Tables 1-2).

**Table 1:** Asymmetric dimethylarginine (ADMA), neopterin (NP), and vitamin D levels of the groups VC +, Vaccinated; VC-, Unvaccinated * p values (< 0.05) indicate statistical significance. ** p values (> 0.05) indicate statistical significance. a: COVID-19 (+) and vaccinated; b: COVID-19 (+) and vaccinated (-); c: COVID-19 (-) and vaccinated (+); d: COVID-19 (-) and vaccinated (-)

Groups	ADMA (µmol/L) (n=30)	NP (nmol/L) (n=30)	Vitamin D (ng/mL) (n=30)
COVID+ VC+^a^	5.60±1.77	3.11±1.29	17.31±6.53
COVID+ VC -^b^	5.05±1.73	2.66±0.59	12.65±4.72
COVID- VC +^c^	4.82±1.87	2.59±1.01	25.04±6.92
COVID- VC -^d^	4.75±0.97	2.17±0.86	21.08±6.15
P value	^a-b**;b-d*;c-d*,a-d*,a-c*^	^a-b*;b-d**;c-d**,a-d+,a-c**^	^a-b*;b-d*;c-d*,a-d+,a-c**^

**Table 2:** Pairwise comparisons of asymmetric dimethylarginine (ADMA), neopterin (NP), and vitamin D levels between the study groups * p values (< 0.05) indicate statistical significance. Arrows (↑ or ↓) show the direction of change in the first group compared to the second.

Comparison Groups	ADMA	NP	Vitamin D	Statistical Significance
COVID+ VC+ vs COVID+VC-	0.761	0.010*	0.010*	NP ↑, Vit D ↑
COVID+ VC- vs COVID−VC-	0.001*	0.220	0.010*	ADMA ↑, Vit D ↓
COVID− VC+ vs COVID−VC-	0.002*	0.565	0.010*	ADMA ↑, Vit D ↑

The highest NP level was found in the COVID+/vaccinated+ group (3.11 ± 1.29 nmol/L), while the lowest was found in the COVID-/unvaccinated group (2.17 ± 0.86 nmol/L). Comparisons using the Kruskal-Wallis test revealed statistically significant differences between the groups (p<0.05). This finding suggests that COVID-19 infection may increase inflammation and that vaccination may modulate this effect (Tables 1-2).

Vitamin D levels

The highest levels were observed in the COVID-19/vaccinated+ group (25.04 ± 6.92 ng/mL), while the lowest levels were observed in the COVID-19/unvaccinated group (12.65 ± 4.72 ng/mL). Comparisons using the Mann-Whitney U test revealed statistically significant differences between the groups (p<0.05). This suggests that COVID-19 infection may negatively impact vitamin D levels, and vaccination did not significantly alter this effect (Tables 1-2).

**Figure 1:**
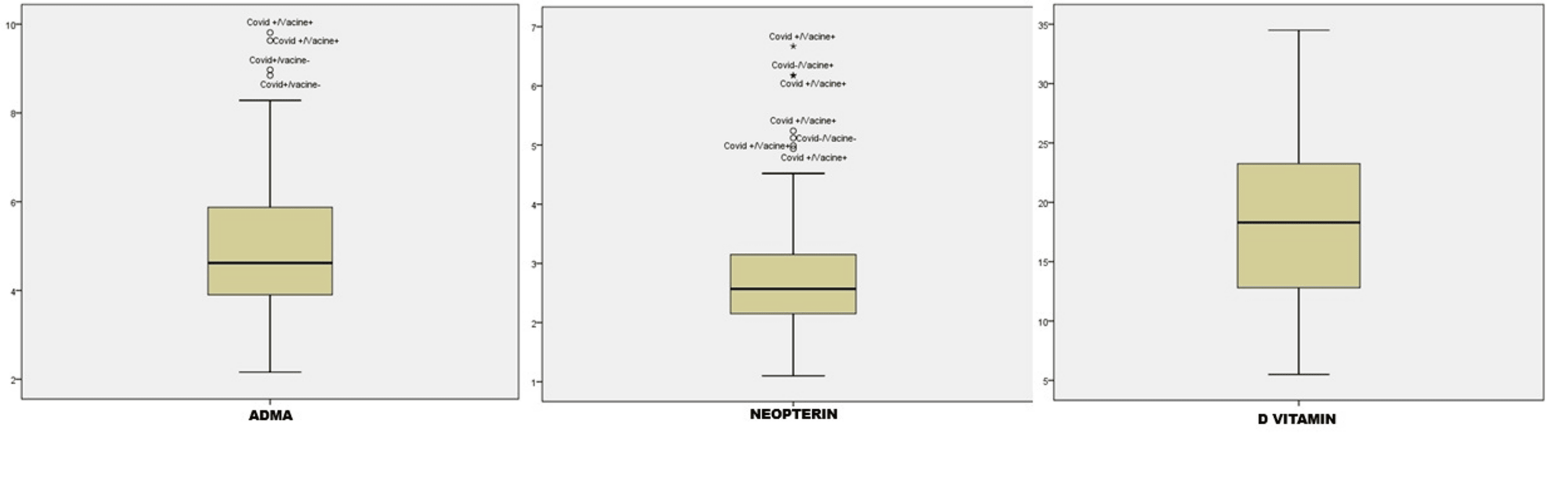
Histogram of the distribution of the study data in the groups

Furthermore, the low, but not significant, vitamin D levels observed in COVID-19-positive participants are considered a significant finding (Table 1 and Figure 1). It is also noteworthy that vaccination did not significantly affect ADMA levels in COVID-19-negative individuals (p=0.559; p>0.05), but vaccination did lead to a statistically significant change in NP levels (p=0.021). However, higher vitamin D levels were observed in vaccinated individuals compared to unvaccinated individuals, and this was statistically significant (p=0.010; p<0.05) (Table 1 and Figure 1).

Pairwise comparisons

When pairwise comparisons of ADMA, NP, and vitamin D levels were examined separately across all study groups, it was observed that the change in ADMA levels was significant depending on COVID+ status, regardless of whether the vaccine was administered or not, and was not statistically significant between the COVID+VC+ and COVID+VC- groups. When the COVID variable was held constant, changes in NP and vitamin D levels varied according to vaccine administration and were particularly statistically significant between the COVID+VC+ and COVID+VC- groups, and the COVID-VC+ and COVID-VC- groups (Tables 1-2).

## Discussion

COVID-19 infection has been associated with severe clinical progression and increased mortality risk, particularly in individuals with chronic cardiovascular diseases [6]. It poses serious risks, especially in young adults under 40 years of age, by affecting many pathophysiological pathways [25,26]. Elevations in ADMA, a key marker of endothelial function, and NP, an indicator of immune response, are linked to increased risk of CAD [8]. Additionally, vitamin D plays a significant role in COVID-19 and cardiovascular health due to its immunomodulatory effects and ability to suppress inflammation [21,27]. COVID-19 vaccination not only reduces the severity of infection but may also influence these biomarkers, thereby playing a role in the evaluation of CAD risk [28]. Therefore, assessing ADMA, NP, and vitamin D levels in the context of COVID-19 infection and vaccination status is crucial for understanding their relevance as risk factors for CAD.

CAD is one of the leading causes of mortality among cardiovascular diseases worldwide. In recent years, numerous studies have investigated the impact of COVID-19 infection and vaccination on the development of CAD. Research focusing on COVID-19 and ADMA levels suggests that this biomarker may serve as a potential indicator for disease progression and prognosis. ADMA is recognized as a marker of endothelial dysfunction and an important parameter for vascular health [29]. In a study conducted by Çetintepe et al. [29], serum ADMA levels were found to be significantly lower in patients with COVID-19 pneumonia compared to the control group (p=0.042). Blood samples were collected in the morning within the first three days of hospital admission. Furthermore, the study reported a mortality rate of 21%, with mortality significantly associated with elevated SOFA and qSOFA scores [29].

Similarly, Haşimi et al. [30] demonstrated that serum levels of ADMA, symmetric dimethylarginine (SDMA), and n-monomethyl-1-arginine (L-NMMA) were correlated with COVID-19 severity. Their findings indicated that patients with severe COVID-19 exhibited significantly higher serum ADMA, SDMA, and L-NMMA levels compared to those with mild to moderate disease and healthy controls (p<0.001). Additionally, L-NMMA, C-reactive protein (CRP), and neutrophil-to-lymphocyte ratio (NLR) were identified as independent risk factors for predicting disease severity [30].

There is a limited number of studies investigating the effect of COVID-19 vaccination on ADMA levels in individuals. Although no specific study has directly evaluated the impact of vaccination on ADMA levels, it is generally known that COVID-19 vaccination reduces disease severity and attenuates inflammatory responses. In this context, it is plausible that vaccination may indirectly influence ADMA levels.

In our study, a statistically significant increase in ADMA levels (% ± SD) was observed in individuals who had recovered from SARS-CoV-2 infection (p=0.001; p<0.05). This finding was supported by ANOVA tests in group comparisons, indicating a significant difference between groups overall (p<0.05). ADMA is accepted as a marker of endothelial dysfunction and plays a critical role in vascular inflammation. Systemic inflammation caused by COVID-19 can elevate ADMA levels through pathways such as macrophage activation via IFN-γ and oxidative stress [30,31]. Karacaer et al. demonstrated that ADMA levels were significantly higher in patients with severe COVID-19 compared to those with moderate disease (p=0.012) [32]. These findings further support our hypothesis that ADMA could serve as a predictive biomarker for COVID-19 prognosis. In our study, no significant change in ADMA levels was detected in vaccinated individuals who had not contracted COVID-19 (p=0.559; p>0.05). This result may indicate that SARS-CoV-2 vaccines do not induce persistent endothelial dysfunction or have lasting effects on ADMA. Although literature on the effect of vaccination on ADMA is limited, some findings suggest that general physical primary immunizations might slightly increase ADMA levels (p=0.001) [33]. Therefore, our data support the hypothesis that COVID-19 vaccines do not trigger systemic inflammation or maintain it within controllable limits. A study supported by NHLBI/NIH demonstrated that SARS-CoV-2 can directly infect coronary arteries, causing severe inflammation in plaques and increasing the risk of myocardial infarction and stroke [34]. Since the beginning of the pandemic, it has been established that COVID-19 increases mortality and the risk of acute coronary syndrome in patients with cardiometabolic comorbidities, such as diabetes and hypertension. A prospective cohort study including 180 patients conducted across four university hospitals in Germany showed that serum ADMA and SDMA levels were significantly higher in COVID-19 patients compared to healthy controls; elevated levels were found to be strong predictors of in-hospital mortality [35].

Furthermore, the meta-analysis by Ye et al. [36] demonstrated that high ADMA levels were associated with a 2.10-fold (95% CI: 1.46-3.02) increased risk of all-cause mortality, 2.49-fold (95% CI: 1.34-4.65) cardiovascular mortality, and a 1.71-fold (95% CI: 1.27-2.32) increased risk of major adverse cardiovascular events (MACE) in patients with CAD [36]. Bellmann-Weiler et al. reported that serum NP levels above 45 nmol/L at hospital admission were significantly associated with severe COVID-19 progression and the need for intensive care [37]. The NP level has emerged as an important prognostic biomarker in evaluating the course of COVID-19. Serum NP concentrations have been associated with the severity and prognosis of SARS-CoV-2 infection. However, studies on NP levels following COVID-19 vaccination are limited. The NP is a compound produced by macrophages upon stimulation with IFN-γ and reflects immune system activation [38].

In a cohort study involving 34 patients, Robertson et al. [39] reported that the mean serum NP level was 42.0 nmol/L in severe COVID-19 cases, compared to 16.9 nmol/L in mild cases; this difference exceeded the upper reference limit of 9.1 nmol/L and was statistically significant (p<0.001). These findings demonstrate a strong correlation between infection severity and NP levels [39]. Conversely, another study involving hospitalized COVID-19 patients found no statistically significant difference in serum NP levels between vaccinated and unvaccinated groups; only urinary NP levels showed a slight difference (p=0.031), which may not have direct clinical significance [40].

The NP production is activated via IFN-γ-mediated macrophage stimulation, reflecting antigen presentation, monocyte-macrophage activation, and Th1 cell-mediated immune responses [39]. In our study, a significant increase in NP levels was observed in vaccinated individuals without prior COVID-19 infection. This suggests that post-vaccination immune response can induce a mild inflammatory effect via antigen presentation without reaching the levels seen in severe disease profiles. Previous studies have shown that NP levels correlate with COVID-19 severity. For example, one study found mean serum NP levels of 42.0 nmol/L in severe COVID-19 patients and 16.9 nmol/L in mild cases. Moreover, NP levels remained elevated at disease onset in severe patients and declined over time [39]. Another study reported that high NP levels at hospital admission were significantly associated with intensive care unit (ICU) admission, mechanical ventilation requirements, and mortality risk [37]. In our study, NP levels increased in individuals who had recovered from COVID-19; however, this increase was not statistically significant (p=0.847). Vaccination in COVID-19 naïve individuals showed a statistically significant effect on NP levels (p=0.021). Group comparisons via Kruskal-Wallis revealed statistically significant differences (p<0.05), suggesting that COVID-19 infection may increase inflammation and vaccination modulates this effect. The vaccination-induced increase in serum NP levels (p=0.021) and significant differences among groups (p<0.05) indicate that NP may serve not only as a marker of COVID-19 severity but also of vaccine-induced immune activation. However, this increase should be interpreted as limited immune stimulation due to antigen presentation from vaccination and not equated with the inflammatory burden seen in infection-associated pathology. Although some cardiovascular studies have suggested that NP might inhibit endothelial adhesion molecules (ICAM-1, VCAM-1) and thus counteract atherosclerosis, most clinical evidence supports that elevated NP levels are markers of heightened inflammation and increased atherosclerotic risk [41]. Studies conducted during the COVID-19 pandemic have indicated that vitamin D deficiency may increase susceptibility to COVID-19 infection and the risk of hospitalization. Low vitamin D levels have been associated with increased disease severity and complications. A study conducted in the United Kingdom analyzing data from 151,543 participants demonstrated that vitamin D deficiency and insufficiency increased the risk of COVID-19-related hospitalization by 36% and 19%, respectively. However, low vitamin D levels were not found to increase the risk of contracting COVID-19. This study emphasizes the complex nature of the relationship between vitamin D levels and COVID-19 outcomes, which may vary across different populations [42].

In our study, vitamin D levels were found to be lower in individuals who had experienced COVID-19 infection. Among individuals without prior infection, vitamin D levels were higher in vaccinated subjects compared to unvaccinated ones; however, this difference was not statistically significant (p=0.269; p>0.05). Furthermore, the mean serum 25-hydroxy vitamin D levels in individuals infected with SARS-CoV-2 were significantly lower than those without infection. Overall, vitamin D deficiency was frequently observed among infected individuals, consistent with previous meta-analyses reporting that low vitamin D levels in COVID-19 patients constitute a prognostic factor associated with increased disease severity and mortality risk [43]. Conversely, among groups without COVID-19 infection, only a numerical difference in vitamin D levels was observed between vaccinated and unvaccinated individuals, which was not statistically significant (p=0.269). This finding is supported by the literature; for example, a cohort study conducted in Dublin reported vitamin D deficiency prevalence rates of approximately 17.5% and 19.8% in vaccinated and unvaccinated hospitalized COVID-19 patients, respectively, with no significant difference between groups [44]. The effects of vitamin D supplementation on COVID-19 outcomes remain inconclusive. While some studies suggest that vitamin D levels may influence disease progression, others have not established a clear association. Therefore, further high-quality randomized controlled trials are needed to elucidate the role of vitamin D in COVID-19. Vitamin D supplementation may provide potential benefits, particularly for individuals with a deficiency; however, definitive conclusions require additional research.

Limitations

The most significant limitation of our study is the rapid vaccination process, which caused panic among individuals due to the viral disease, causing a pandemic. To ensure balanced and consistent data, equal numbers of individuals were included in the patient groups. This limited sample size and clinical heterogeneity led to a limited number of patients who did not receive vaccination and limited the number of individuals included in other groups. Changes in biomarkers over time may vary depending on the stage of the disease, treatment, and individual factors. This could limit the study. Furthermore, the study was evaluated as a doctoral dissertation and therefore subject to time constraints.

## Conclusions

COVID-19 disease induced a significant increase in ADMA levels in individuals. This increase has been associated with a strong CAD predictor of in-hospital mortality, as seen in most literature in severe COVID-19 cases. Important biomarkers such as ADMA and NP can be considered promising early prognostic tools for assessing the extent of vascular risk in COVID-19. Monitoring these biomarkers may provide a valuable approach to improve coronary risk stratification, particularly in individuals at risk for CAD. Evaluating these parameters in conjunction with vitamin D levels may also contribute to the development of personalized clinical approaches and strategies that enable early intervention. The findings from this study will contribute to insights into the effects of COVID-19 infection and vaccination on biomarker levels and enable early assessment of the relationship of biomarkers with CAD risk.
